# Optimizing Mechanical Structures Through Butt Joining of Dissimilar Materials for Lightweight Components

**DOI:** 10.3390/ma19010018

**Published:** 2025-12-20

**Authors:** Jarosław Szusta, Łukasz Derpeński, Özler Karakaş, Nail Tüzün

**Affiliations:** 1 Department of Machine Design and Exploitation, Faculty of Mechanical Engineering, Bialystok University of Technology, Wiejska 45C Str., 15-351 Bialystok, Poland; j.szusta@pb.edu.pl (J.S.); l.derpenski@pb.edu.pl (Ł.D.); 2 Mechanical Engineering Department, Engineering Faculty, Pamukkale University, Kinikli, 20160 Denizli, Türkiye; 3 Department of Mechanical Engineering, Engineering Faculty, Istanbul Arel University, Tepekent, Buyukcekmece, 34537 Istanbul, Türkiye; nailtuzun@arel.edu.tr

**Keywords:** dissimilar welding, lightweight structures, mechanical structure optimization, CMT welding, high-strength steel

## Abstract

The joining of dissimilar steels is crucial for designing lightweight, high-performance structures but poses significant challenges due to uneven material properties. This study optimizes the butt-welding process for a dissimilar pair of S355J2 and Strenx 700E steels. Cold Metal Transfer welding was employed, and the effects of surface preparation, linear energy, and joint gap on joint integrity were systematically investigated via tensile testing, digital image correlation, fractography, and microhardness analysis. The results demonstrate that mechanical surface cleaning combined with a low linear energy of 0.334 kJ/mm and a 0.5 mm gap yields optimal performance. This parameter set produced a joint with a tensile strength of 616 MPa, representing a 32% increase compared to uncleaned samples, and promoted uniform plastic deformation across the joint. Microstructural analysis confirmed a narrower heat-affected zone and the absence of significant softening in the high-strength steel.

## 1. Introduction

In an era of raw material crisis and high tariffs on imported materials, it is becoming increasingly important to combine dissimilar materials of widely varying mechanical strength to create more lightweight, stronger, and efficient structures. The main motivations stem from the need to optimize weight and cost while maintaining the required strength and safety properties of the structure. Examples include combinations of low-carbon or unalloyed steels with high-strength steels with improved mechanical resistance, such as bainitic, martensitic, or Ultra High Strength Steel (UHSS). The process of joining these materials, as opposed to welding materials of the same grade, is complex. It requires a precise selection of methods, parameters, and an understanding of the properties of each material to be joined in order to achieve optimal joint strength. Beyond civil engineering and automotive applications, sectors such as heavy machinery and commercial transportation are increasingly demanding lightweight yet durable mechanical structures. The demand for lightweight yet durable structures is particularly acute in sectors such as commercial vehicles, heavy machinery, and load-bearing equipment. In these applications, the integrity of every joint is important, as it directly dictates the overall structural performance, safety, and efficiency. Consequently, the capability to reliably weld dissimilar steels has allowed for weight and cost optimization while unlocking new possibilities in advanced manufacturing. Therefore, the outcomes of this study contribute to optimizing structural joining methods that can be directly implemented in advanced manufacturing technologies, particularly in the fabrication of robust structures, protective housings, and lightweight support frames. This paper focuses on the analysis of the different parameters of industrial welding, their influence on the quality of the joints, and the possibilities of optimizing this process in the context of high-volume production.

With the intensive development of engineering technologies and increasing demands for energy efficiency, durability, and sustainability, optimizing the weight of mechanical structures is becoming one of the key design goals. Reducing the weight of a structure while maintaining or improving its performance requires the use of advanced materials, including joining them in dissimilar configurations. Welding, as one of the most common methods of permanently joining metals, poses a significant technological challenge when materials with different physical and chemical properties, such as thermal conductivity, coefficient of thermal expansion, crystal structure, or chemical composition, are involved.

Research into the selection of optimum joining parameters for dissimilar materials and their effect on strength has taken center stage in recent years. Numerous studies [1,2,3] in this area focus on the selection of suitable welding methods that will provide durable and strong joints while minimizing the risk of defects or weakening of the structure of the materials being joined. According to the literature, one of the most commonly used processes is arc welding, including TIG and MIG, which allow for precise joining of different metals. Studies presented in [4,5,6,7] have shown that the appropriate choice of welding parameters significantly affects the quality of the joint, especially for materials with different physical and chemical properties. Another important aspect of the joining process of dissimilar materials is the problem of thermal divergence between materials, which can lead to internal stresses and cracks. Works [8,9] highlights that the use of special techniques, such as laser welding or welding with filler materials, can help to minimize these problems. There are also studies in the literature on modern technologies, such as ultrasonic welding or hybrid welding, which open up new possibilities in joining dissimilar materials. For example, the information presented in the work [10,11,12] shows that the use of these methods can significantly improve the quality and durability of the joints, as well as enable more efficient mass production.

Joining dissimilar materials, e.g., those that differ significantly in yield strength, carries with it a number of problems, including the formation of brittle intermetallic phases, uneven stress distributions, and microstructural compatibility issues. As indicated by works [13,14], joining steels with significantly different strength parameters—e.g., S235JR steel (tensile strength ~360 MPa) and DP1000 steel (strength ~1000 MPa) results in non-uniform stress distributions, an asymmetric heat-affected zone (HAZ), significant differences in hardness on the weaker material side, and the risk of cold or hot cracking within the joint.

Microstructural studies show that differentially eccentric steel joints often develop a soft zone in the HAZ on the higher strength steel side, which can lead to a reduction in local joint strength [8]. The high stress concentration in this zone can result in fatigue crack initiation, which limits the use of such joints in dynamically loaded structures.

Increasingly, low-energy, precise, and more controlled techniques such as laser welding [10,15] friction stir welding (FSW) [16], and hybrid welding (laser + MAG) [13,17] are being used to weld materials with dissimilar properties. Among advanced gas metal arc welding (GMAW) variants, Cold Metal Transfer (CMT) has gained significant prominence for joining dissimilar and thin materials. Its fundamental process characteristics, such as controlled material deposition, inherently low heat input, and reduced spatter, make it particularly suitable for overcoming challenges like excessive dilution, distortion, and crack sensitivity in dissimilar joints [18,19,20,21]. Furthermore, the versatility of the CMT process is evidenced by its exploration in and integration with advanced manufacturing contexts, such as directed energy deposition (DED) [22,23]. However, the vast majority of industrial plants producing mechanical structures use traditional gas-shielded welding methods. Therefore, there is a justified need for research to select optimal joining parameters for these materials, even when using advanced processes like CMT.

## 2. Materials and Methods

### 2.1. Base Materials and Properties

In the manufacture of various steel structures for which weight optimization is required, a common combination of dissimilar materials is steels with significantly different mechanical properties, particularly where the ultimate strength differs by at least a factor of two. This study focuses on the butt joint between S355J2 steel (Thyssenkrupp AG, Essen, Germany) and Strenx 700E steel (SSAB, Stockholm, Sweden), a material combination previously investigated for overlay joints in lightweight applications [24]. This combination requires a specific approach to the joining process due to the twofold difference in yield strength between the two materials. Combining the low-cost structural steel S355J2 with the high-strength steel Strenx 700E enables the production of stronger, lighter, and more fuel-efficient machines with an extended service life.

The strength properties and chemical composition of the base materials are detailed in Table 1 and Table 2, respectively.

### 2.2. Joint Design and Specimen Preparation

For weight-optimized structures where joined elements are of constant width, a method for selecting the thickness of dissimilar materials is proposed to design uniformly stressed structures. The thickness of the higher-strength sheet is determined based on the yield strengths and the thickness of the lower-strength sheet according to the Equation (1).

(1) $$g_{H}=\frac{R_{eL}}{R_{eH}}g_{L}$$

where *g*_H_ [mm] is the thickness of a higher strength sheet, *g*_L_ [mm] is the thickness of a lower strength sheet, *σ*_YL_, *σ*_YH_ are the yield strength of the lower and higher strength material respectively, given in [MPa].

**Figure 1:**
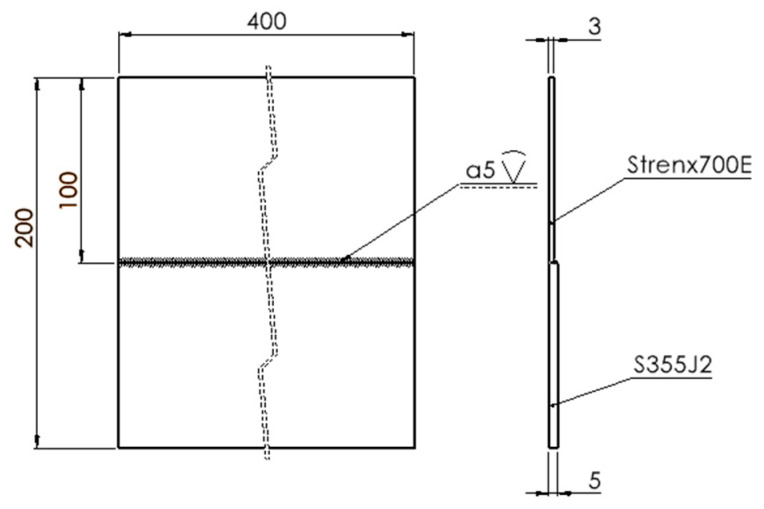
Butt welded joints made of dissimilar materials.

Based on this relationship, the base material was structural steel S355J2, with a thickness of 5 mm, butt-welded to a 3 mm thick plate made of Strenx 700E. The geometry of the butt-welded joints is shown in Figure 1.

In order to carry out the planned activities, test joints were made from dissimilar materials. The base material was ordinary S355 structural steel with a thickness of 5 mm, butt-welded to a 3 mm thick sheet made of Strenx 700E material. The welds were made using CMT technology. A welding electrode in the form of Lincoln Supramig HD ISO 14341-A-G46 4 M21 [25] wire (Lincoln Electric Bester Sp. z o. o., Bielawa, Poland) was used for welding. The welding process was carried out using the parameters listed in Table 3. First, both materials, which had mill scale contamination on their surfaces, were joined together. Then, the same joint was made by mechanically cleaning the joined elements. Different welding parameters were used for welding. In the next approach, joints were prepared in which the elements were spaced 1 mm apart.

The linear welding energy was determined from the Equation (2):

(2) $$Q=\frac{IU}{V\;1000}k\;[\frac{kJ}{mm}]$$

where *I*—average value of welding current [A], *U*—average value of arc voltage [V], *V*—welding speed [mm/s], *k*—thermal efficiency coefficient (for the parameters used *k* = 0.8).

The following assumptions were made when performing the test joints: for the first configuration of the samples, the surface of the workpieces to be joined was cleaned by shot-blasting (samples S1, S2, S3); the second configuration was raw material without pre-cleaning the surface before welding (samples S4, S5, S6, S7). Different welding parameters were used for both configurations. For samples S1 to S6, a gap of 0.5 mm was used and the welding torch was inclined towards the thinner material to be joined, while for sample S7 the joined parts were set with a gap of 1mm. The study was intended to determine the effect of preparatory welding operations on the strength of dissimilar materials that could be used for structural frame structures and to allow technological, process, and economic optimization of the joints made.

**Figure 2:**
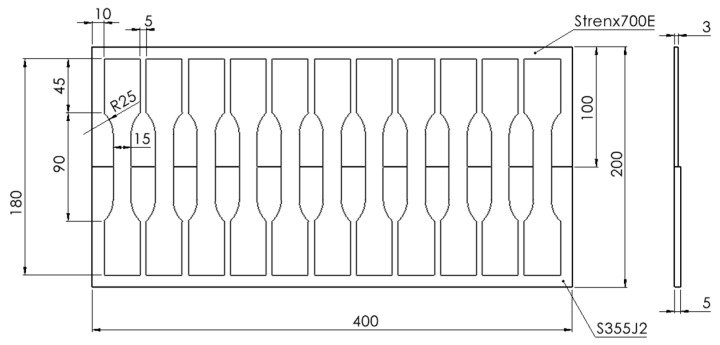
Template for the placement of paddle specimens on a prepared butt weld joint.

**Figure 3:**
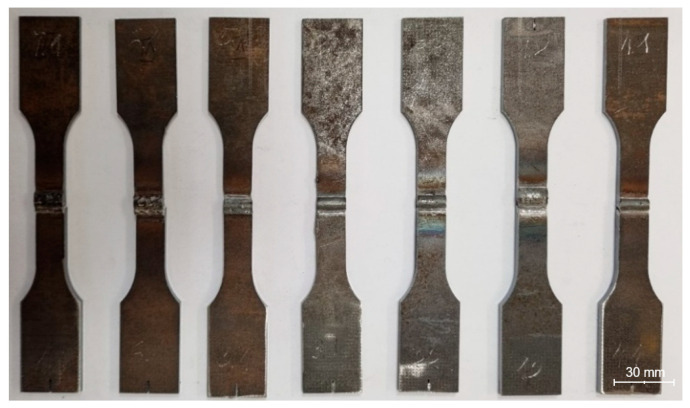
Views of cut paddle samples.

For the produced test joints, standard paddle specimens were extracted using plotter cutting technology according to the template shown in Figure 2. The reduction in specimen weight by using this dissimilar material combination, compared to a specimen made entirely of S355J2, was 20%. Photographs of resulting test samples were presented in Figure 3.

### 2.3. Test Stand

**Figure 4:**
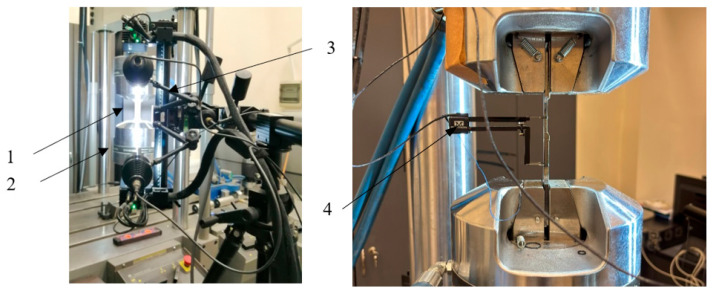
Sample on test stand: 1. samples; 2. testing machine grips; 3. DIC Aramis; 4. Extensometer Instron.

The sample was loaded kinematically at a constant speed of Δl = 0.01 mm/s set via an Instron linear extensometer with a 50 mm measuring base. The specimen (1) on the test stand is shown in Figure 4. An MTS 828 servohydraulic testing machine (MTS Systems Corporation, Eden Prairie, MN, USA) (2) was used for the tests. During the test, the force and displacement of the specimen’s load base were recorded continuously. In addition, the deformation of the specimen during load build-up was observed using the digital image correlation (DIC) method with the ARAMIS 4M vision system (Carl Zeiss GOM Metrology GmbH, Braunschweig, Germany) (3). This made it possible to record the deformation process and to locate critical deformation zones causing the failure of the specimen.

The aim of the tests was to determine the strength characteristics of the welded joints and to determine the deformation distributions in the analyzed test samples. Each test was repeated three times, and the result was the arithmetic mean of the three repetitions.

These tests consisted in determining the impact of welding techniques and parameters on the mechanical properties of the joint, including the maximum tensile force (Fmax), the force causing plastic deformation in the sample (FRE), and the maximum elongation of the sample to break (A%).

In addition, the fracture of the samples was analyzed to detect weld defects such as cracks, undercuts, lack of fusion, slag, and bubble pockets. Based on the observation of the fracture site, the correctness of the joined materials and the selection of welding parameters was determined.

### 2.4. Microhardness Measurements

In order to assess changes in the mechanical properties of the joints, Vickers microhardness (HV_0.05_) measurements were performed on cross-sections of the samples. Fragments of individual joints were cut in the plane of the joint, ground on a set of 3000-grit sandpaper, and then polished using diamond abrasive. The samples prepared in this way were etched using ADLER reagent. The microhardness of the joint was measured at a constant step. The tests were performed at a load of 4.9 N for 15 s. The measurements were taken from the weld axis in both directions, assuming a distance of 1 mm between points with the measurement line in the middle of the thickness of the thinner of the joined materials.

### 2.5. Fracture Topography Observations

The fracture morphology was analyzed using a Keyence VHX-X1 digital microscope (Keyence Corporation, Osaka, Japan), performing observations at various magnifications in order to identify the characteristic features of the fracture surface. The samples for observation were prepared by cleaning the fracture surface with isopropyl alcohol and drying in air. The topography of the fractures, the location of crack initiation and the proportion of ductile and brittle zones were documented. The recorded images were used to assess the failure mechanism and verify the locations of crack initiation in relation to the deformation field distribution. Additional observations of the fracture topographies were carried out using a scanning microscope Phenom XL (Thermo Fisher Scientific Inc., Waltham, MA, USA) at 300× and 2500× magnification. The test specimens were taken from the fracture location, and after cutting and blowing with compressed air, the surfaces were degreased with acetone. The observations were made in the middle of the working width of the specimen.

The methodology used enabled a comprehensive analysis of the influence of welding parameters on the temporary strength of a dissimilar joint subjected to uniaxial tension. The combination of mechanical signal recording, visual deformation measurements, microhardness analysis and microscopic observations allowed for a complete characterization of the deformation processes and crack locations in the butt joint zones.

## 3. Results and Discussion

### 3.1. Monotonic Tensile Tests

**Figure 5:**
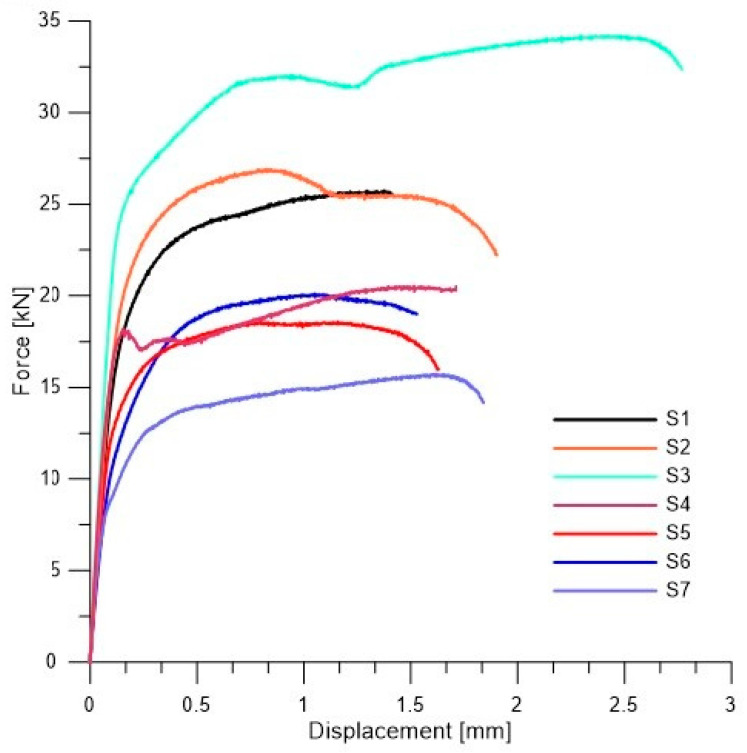
Monotonic tensile force-displacement curves of the analyzed specimens.

Firstly, welded joints of dissimilar materials made with different parameters were tested. The monotonic tensile curves from the tests performed are shown in Figure 5.

Considering that the material joints analyzed are components of different thicknesses, it was assumed for the purposes of comparison that the values of forces were related to the average cross-section of the specimen when determining the approximate stresses. The research presented in this paper is a comparative analysis of technological variants used industrially in CMT welding of dissimilar materials. They refer to the determination the strength of the joint in uniaxial tensile testing of paddle samples. When determining the apparent parameters of the joints, it was assumed that the engineering stresses would be averaged by relating the force value to the average cross-section of the joint. This allowed for a comparative analysis of welded joints made with different parameters.

**Figure A1:**
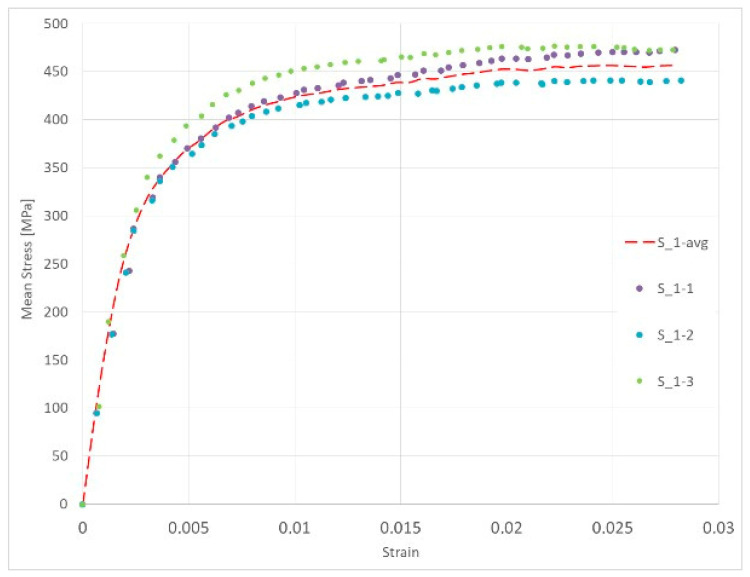
Monotonic tensile curves of Sample S1.

**Figure A2:**
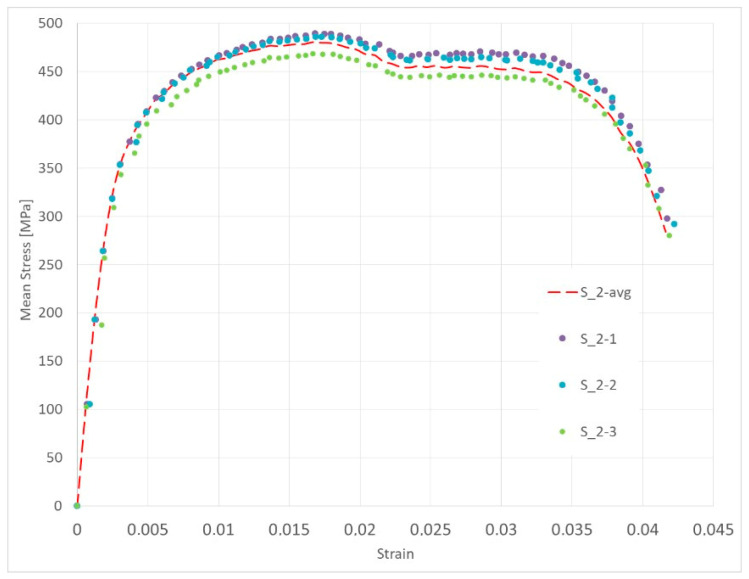
Monotonic tensile curves of Sample S2.

**Figure A3:**
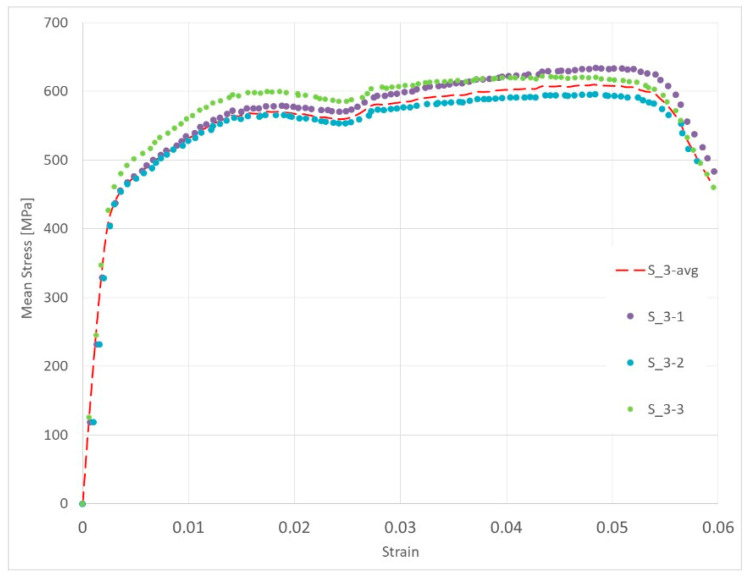
Monotonic tensile curves of Sample S3.

**Figure A4:**
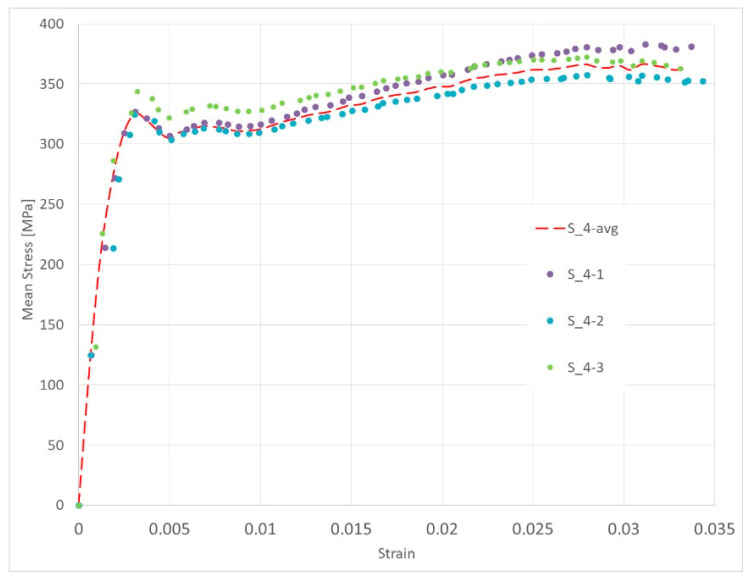
Monotonic tensile curves of Sample S4.

**Figure A5:**
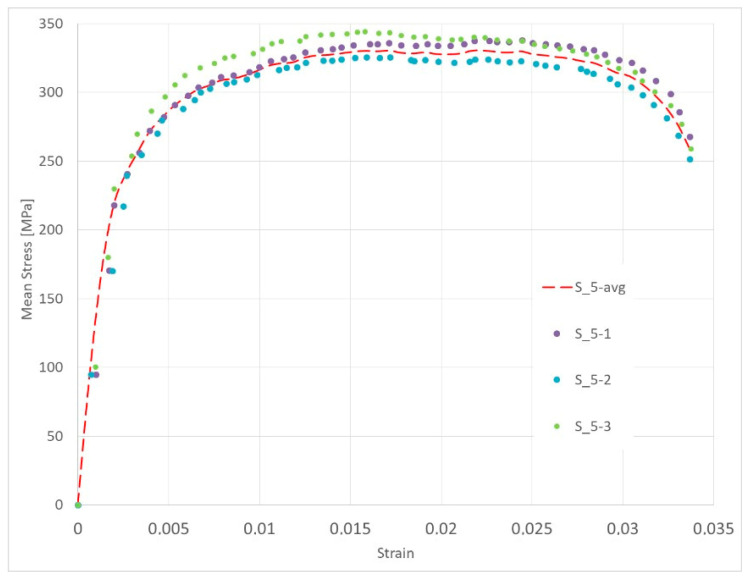
Monotonic tensile curves of Sample S5.

**Figure A6:**
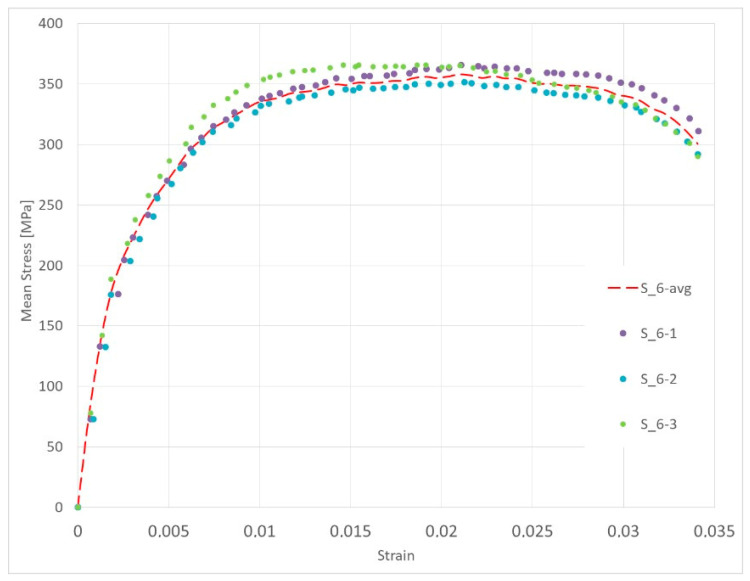
Monotonic tensile curves of Sample S6.

**Figure A7:**
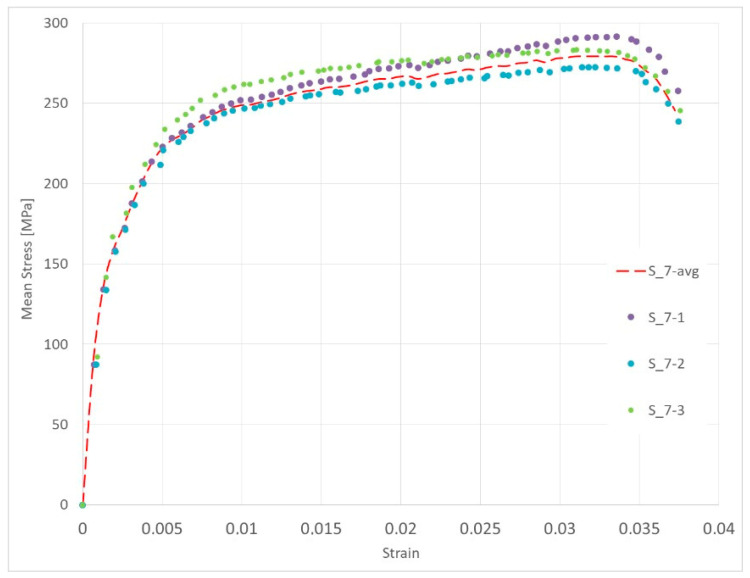
Monotonic tensile curves of Sample S7.

Additionally detailed test results of each specimens were provided in Appendix A (Figure A1, Figure A2, Figure A3, Figure A4, Figure A5, Figure A6 and Figure A7). The parameters describing the analyzed welded joint, determined according to the assumptions made, are presented in Table 4. They were calculated as the arithmetic mean of three repetitions. The tensile curves show the response of the welded joint to a kinematically applied load. Due to the averaging of the cross-section of the sample, they should be treated as welded joint curves rather than material curves.

**Figure 6:**
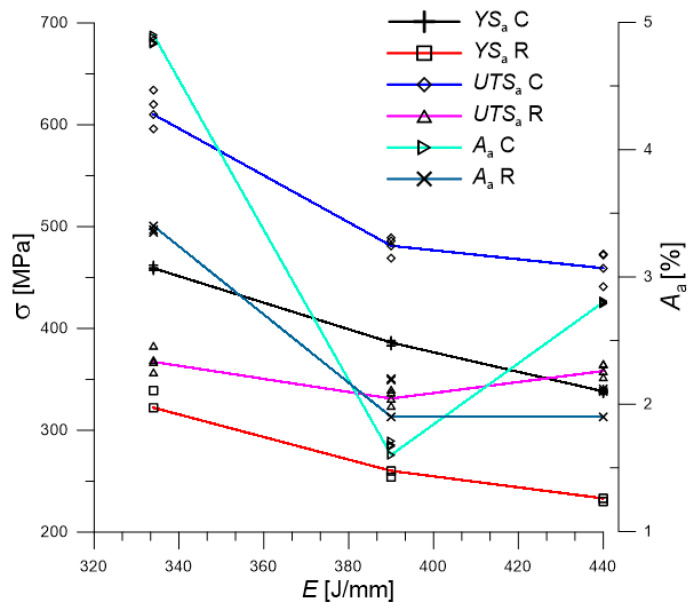
Influence of welding line energy on the parameters of a dissimilar butt joint; *C*—samples cleaned before welding; *R*—raw samples before welding.

Analyzing the data obtained (Figure 6), it can be concluded that with a decrease in welding heat input, both the yield strength of the welded joint and the strength limit itself increase. For the analyzed cases of joints made with prior surface cleaning, this difference in yield strength and strength was 25% on average. In the case of joints made from uncleaned plates, the difference in yield strength was 28%, while for strength only 3%.

The observed inverse relationship between linear energy and joint strength is likely attributable to the resultant microstructure. Lower heat input typically results in a faster cooling rate, which can refine the grain structure in the weld metal and HAZ, resulting in an increased yield strength. Furthermore, reduced heat input minimizes the width of the HAZ and the extent of the “softened” area on the high-strength steel side (Strenx 700E). These observations are further supported by microhardness measurements, presented later in the paper.

Comparing the results obtained for the same welding parameters and different qualities of surface preparation for welding (cleaning and no cleaning), it is found that, on average, a joint made without pre-weld surface preparation withstands 68% of the load tolerated by joints with cleaned surfaces before welding can carry.

**Figure 7:**
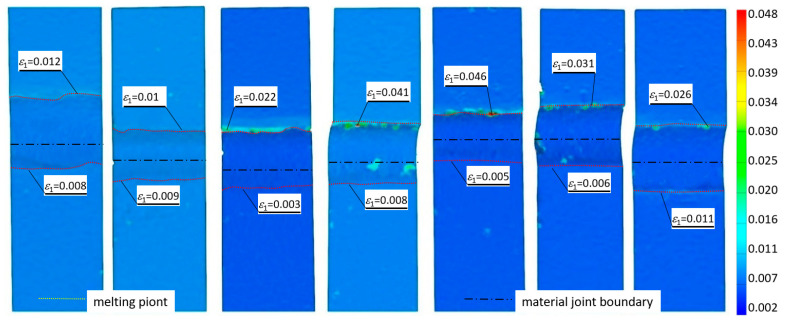
Tensile strain distributions of welded joints were recorded for the load level corresponding to the yield point.

**Figure 8:**
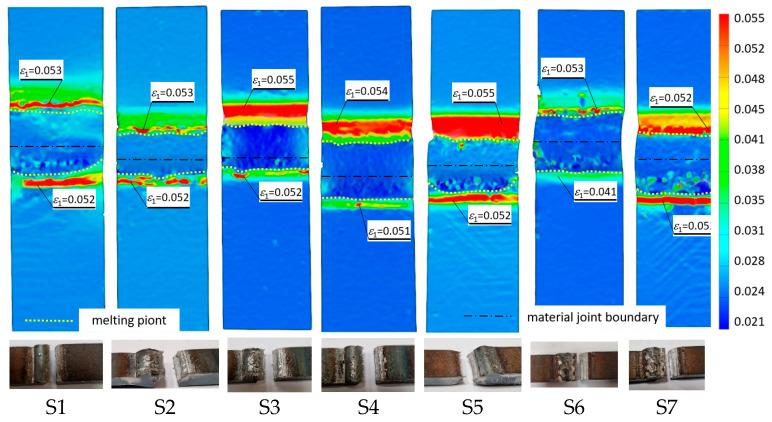
Tensile strain distributions of welded joints were recorded for the load level corresponding to the strength limit and the breakthroughs obtained during the test.

Figure 7 and Figure 8 show the strain distributions recorded for the test specimen types analyzed for load levels corresponding to yield and maximum tensile loads.

The results of the study of the recorded deformations at the joining interface of the two structurally different materials show that there is a clear effect of the quality of surface preparation of the materials to be joined, their correct positioning during welding, and the amount of heat introduced into the materials. A low welding heat input increases the ductility of the joint, resulting in a uniform plasticization of the material of the samples (Figure 7). Similarly, the joints exhibited behavior under loads corresponding to their strength limit (Figure 8, samples S3, S4, and S5 obtained a wider zone of plastic deformation than the others). For this load level, the lower strength material (S355J2 steel) shows bands of slip coming to the surface, visualized as oblique bars. For Strenx 700E steel, this phenomenon is not observed in the made joints, regardless of the parameters used for its preparation.

### 3.2. Weld Geometry Analysis

In the next step, the structure of the welded joint was analyzed in order to determine the characteristic dimensions of the joint zones. The joints made of dissimilar materials were subjected to metallographic testing to reveal the characteristic zones of the joint. The specimens were subjected to grinding, polishing and an etching process in the chemical reagent Adler 2 before the basic measurements. Table 5 shows the results obtained during the analysis.

In the case of samples cleaned before welding, the fusion area increases evenly with the increase in welding linear energy. For uncleaned samples, the size of this area also increases, but unevenly. The situation is similar in the case of the HAZ for cleaned samples for both joined materials. In uncleaned samples, the HAZ shows greater variability. At the lowest linear welding energy (334 J/mm), raw samples have greater penetration than cleaned samples. This may be due to local contaminants (oxides, lubricants) that modify the CMT arc (e.g., local arc concentration, coating penetration), leading to uneven penetration. At medium energy (390 J/mm), the differences in the penetration field between clean and raw samples disappear, but the HAZ in raw samples becomes significantly larger, especially on the S355J2 side. This suggests that impurities change the way heat is distributed (greater heating of the parent material, more extensive overheating). In most cases, the HAZ on the S355J2 side is larger than on the Strenx 700E side under the same welding conditions. This may be due to differences in thermal conductivity, heat capacity and melting point of the alloys (different response to the applied heat input) and different plate thicknesses (5 mm vs. 3mm). The thinner Strenx heats up faster and transfers less energy to deeper zones or undergoes faster remelting, which changes the geometric field of the HAZ.

### 3.3. Strengthening Curves

**Figure 9:**
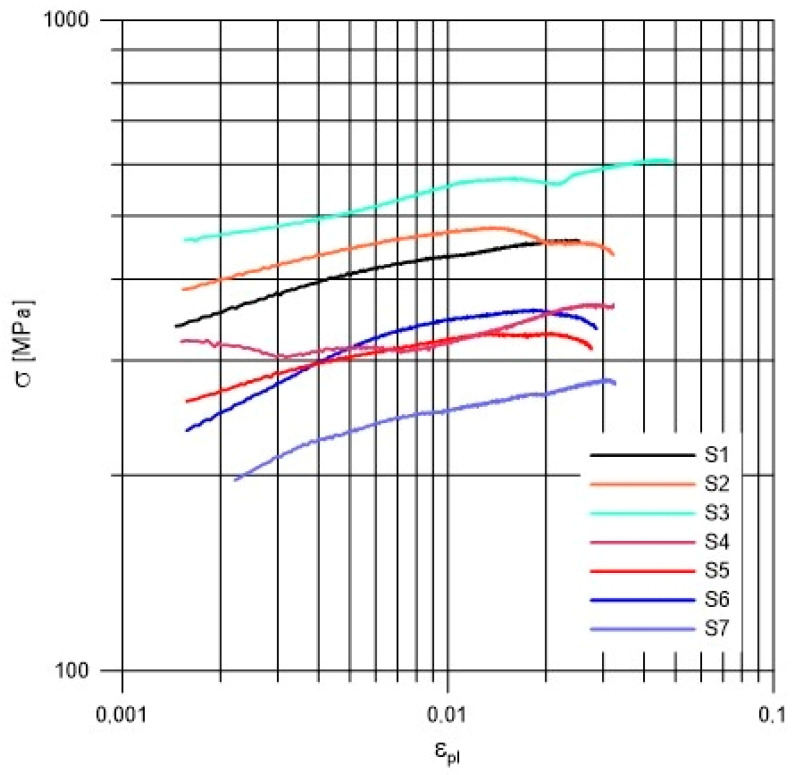
Strengthening curves of the analyzed welded joints.

In order to compare the tested welded joints made of dissimilar materials of different thicknesses, an analysis of the tensile curves of the welded joints in the range of their plastic deformation was performed. The weld tensile curve was divided into a part corresponding to elastic deformation and a part corresponding to plastic deformation in order to obtain complete information about the weld under test. This made it possible to determine the parameters of the weld reinforcement curve (Figure 9). These were obtained by approximating the tensile curves in the range of increasing stresses causing plastic deformation. According to the Ramberg-Osgood relationship presented in Equation (3):

(3) $$\varepsilon =\varepsilon_{el}+\varepsilon_{pl}=\frac{\sigma }{E}+{\left(\frac{\sigma }{K}\right)}^{\frac{1}{n}}$$

The curve of monotonic material strengthening in the logarithmic coordinate system (*ε*_pl_, *σ*) (Figure 9) was approximated by a straight line described, by the Equation (4).

(4) $$\mathrm{lg}\sigma =\mathrm{lg}K+n\mathrm{lg}\varepsilon_{pl}$$

where *σ* is the axial stress, *K* is the strength coefficient, *n* is the strain hardening exponent, and *ε*_pl_ is the corresponding plastic linear strain. The values of *K* and *n* determined by approximating the equation of the welded joint tensile curve, for the joints analyzed, are shown in Table 5.

From the waveforms obtained (Figure 9), it was found that all the specimens tested tended to strengthen as a result of the applied load. The welds carried the load uniformly in most cases, deforming in a manner similar to welded materials. Only on specimens S3 and S4, a slight bump was observed when the specimens were loaded. This may have been due to the release of internal energy stored in the joint during the welding process. However, this did not prevent the joint from reaching its intended strength.

Table 6 shows coefficient and exponent of the monotonic strengthening curve of a welded joint. A comparison of the weld joint parameters obtained for both cleaned (S1, S2, and S3) and uncleaned (S4, S5, and S6) joint surfaces shows that an increase in the *K* factor and exponent *n* of the R-O curve is observed as the welding energy decreases. The opposite is true for joints made on uncleaned surfaces.

For the case of uncleaned surfaces (S4, S5, and S6), the opposite trend was observed, with the values of the R-O parameters taking smaller values than for the cleaned counterparts with smaller parameter values.

Subsequently, the H. N. Hill gain curve parameter of welded joints was determined from the tensile test data using the simplified strengthening curve parameter determination method proposed by H. N. Hill, presented in Equations (5) and (6).

(5) $$\varepsilon =\frac{\sigma }{E}+0.002{\left(\frac{\sigma }{\sigma_{Y}}\right)}^{n_{H}}$$

Hence;

(6) $$n_{H}=\frac{ln\left(\frac{\varepsilon_{max}-\frac{\sigma_{UTS}}{E}}{0.002}\right)}{ln\left(\frac{\sigma_{UTS}}{\sigma_{Y}}\right)}$$

In this case, as the welding heat input decreased, the values of the Hill exponent decreased for both cleaned and uncleaned surfaces. As far as the strength parameters are concerned, however, with a decrease in the material strengthening exponent (Table 7), the strength of the butt welded joint made of dissimilar materials with different strength properties and different thicknesses increases. This fact coincides with the values obtained during the experimental tests corresponding to the strength limit of the joint and confirms the significant influence of the preparation of the welding surface on the strength of the resulting joint.

### 3.4. Fractographic Examinations

**Figure 10:**
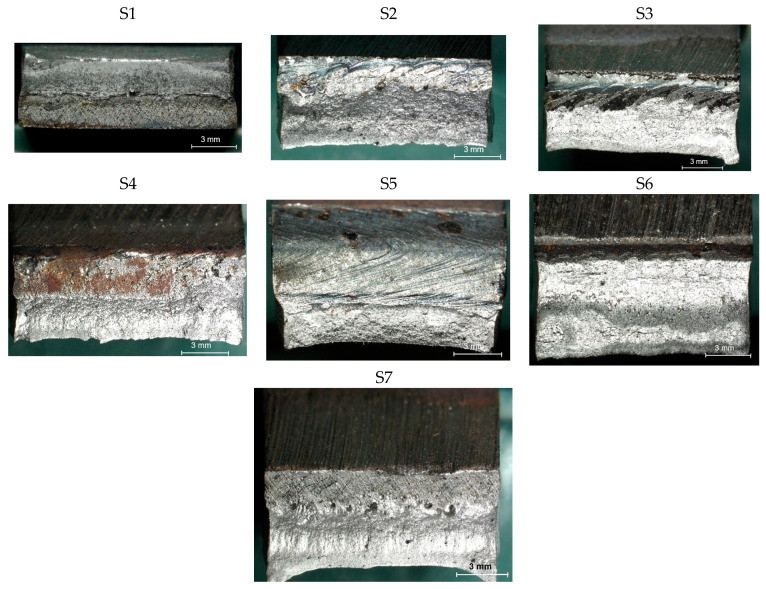
Views of welded joint fractures.

In the next stage of the study, the fracture structure of the welded joint was analyzed. These tests were intended to assess the quality of the weld that forms the joint between the two materials analyzed. Figure 10 shows the results from the analyses carried out.

It was observed that gas bubbles and inclusions (S4, S5, S6, and S7) formed due to the contact of the molten metal with impurities and scale on the surface of the material and were present in the weld on samples that had not been cleaned before welding. The superior strength of sample S3 is corroborated by its fracture surface, which exhibits a mixed-mode failure with areas of ductile dimpling. Conversely, the fractures of samples S4–S7 show features consistent with their lower measured strength: larger, more numerous gas pores and inclusions act as stress concentrators, facilitating brittle initiation and failure. This provides a clear micro-mechanical explanation for the 32% strength reduction observed in uncleaned samples. The voids and foreign inclusions in the material caused a reduction in the adhesion strength of the joint, as observed in the monotonic tensile test plots.

The results of the tests carried out on the recorded deformations at the joining boundary of the two structurally different materials showed that, with a reduction in the amount of heat introduced into the materials in the weld area (samples S3 and S4), an increase in the ductility of the material and a change in the nature of the fracture to a more ductile one is observed—a desirable phenomenon for machine operation. In addition, the ductility is higher for samples with cleaned surfaces than for those with scale before welding.

**Figure 11:**
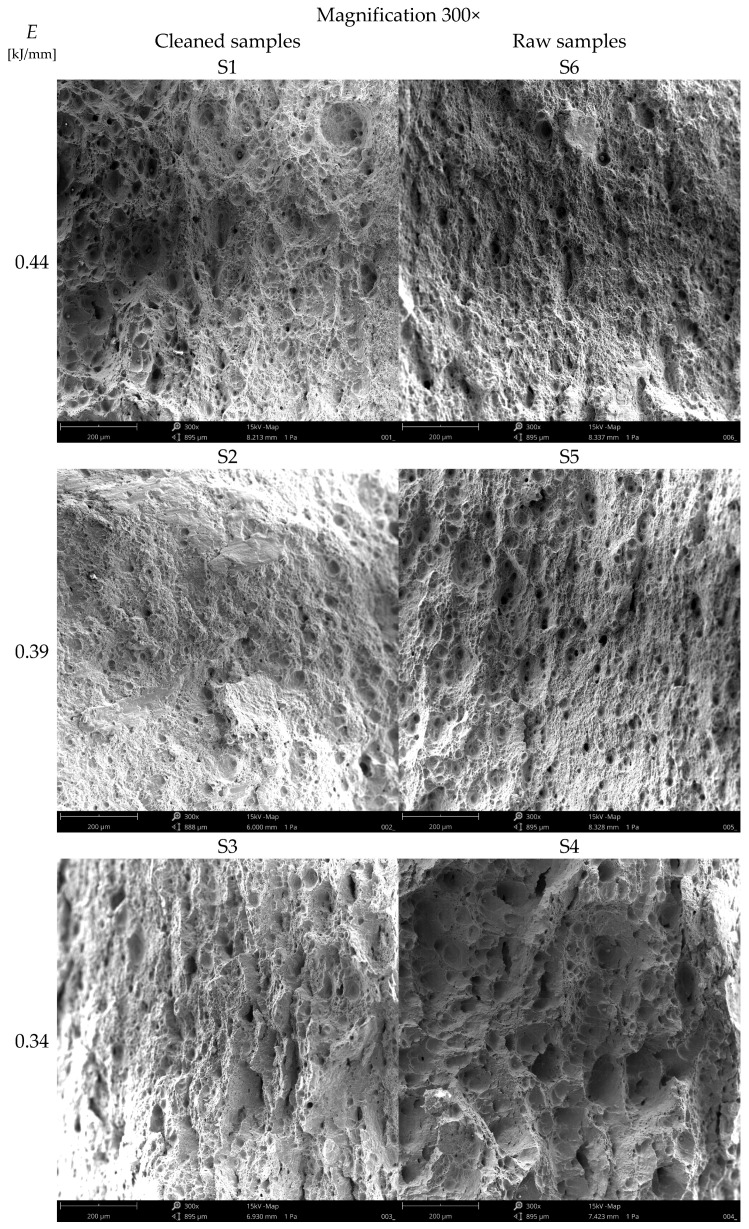

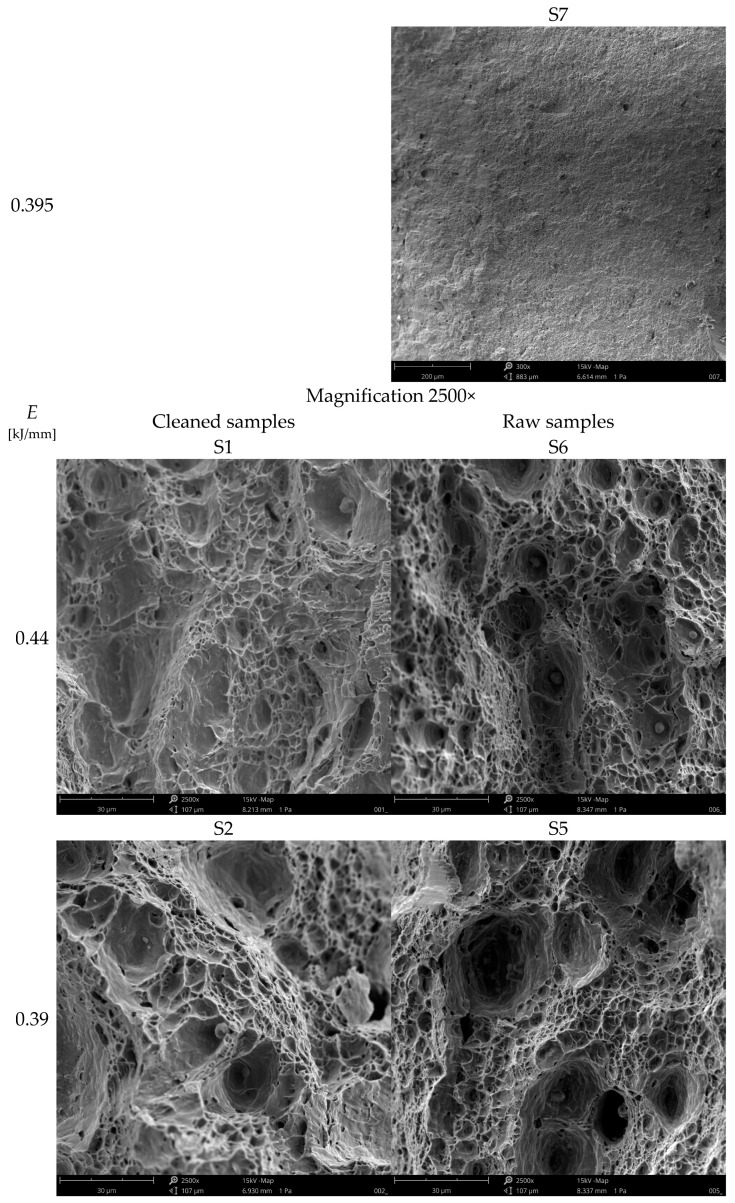

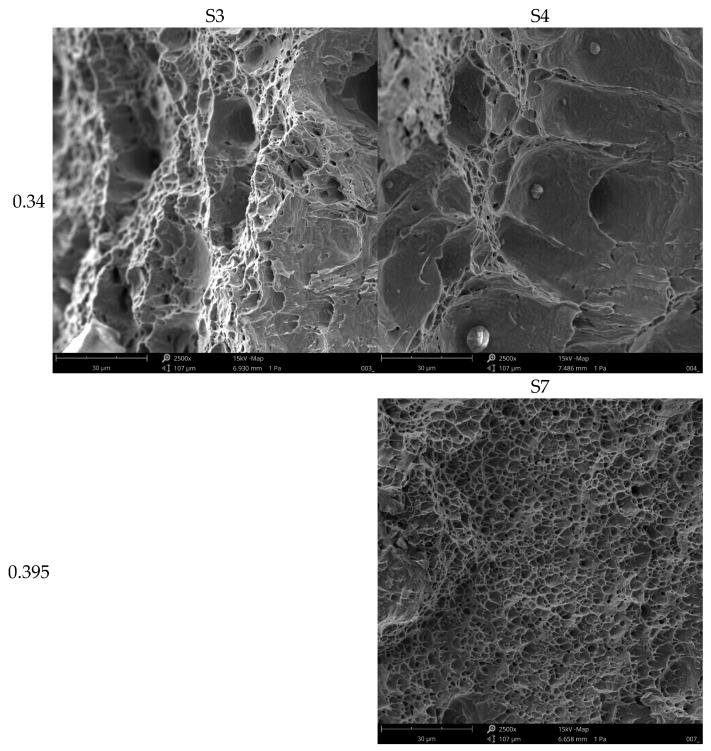
Images of the topography of welded joints obtained at magnifications of 300× and 2500× using a scanning microscope.

Figure 11 shows the topography of welded joints made using the CMT method from dissimilar materials with different welding parameters. Specimens with a cleaned surface (S1–S3) generally show features of more ductile fractures (micro-indentations caused by local plastic deformation) compared to raw specimens (S4–S7), where irregularities indicating inclusions, pores, or local brittle features are more frequently visible (images S1–S3 vs. S4–S7). At higher welding energies (S1, S6—E = 0.44 kJ/mm), greater characteristics associated with more intense heat influence can be seen: smooth surfaces with a visible tendency towards brittle-fracture features. At the lowest energy (S3, S4—E = 0.334 kJ/mm), the image shows planes with sharper edges, fewer traces of plasticity, and fragments with a smooth, quasi-brittle surface, which translates into the strength of the welded joint. These features are more pronounced in raw specimens (S4). This is probably due to the influence of surface contaminants (metallurgical scale) causing stress concentrators and fracture initiation. Specimen S7 (1 mm gap), the fracture pattern indicates variability in weld geometry and greater topographical heterogeneity: both areas with good fusion and areas where the fill is uneven, which correlates with a larger initial gap and more difficult control of the melt shape.

The fracture morphology and mechanical integrity of the welds were significantly influenced by surface preparation, welding energy, and gap geometry. Specimens with cleaned surfaces exhibited fewer inclusions and a morphology dominated by micro-indentations and bulges, indicative of plastic fracture, whereas raw surfaces contained embedded contaminants that served as initiation sites for brittle fractures. With regard to welding energy, a high energy input (0.440 kJ/mm) promoted better fusion but at the same time increased the thermal impact leading to a fracture morphology dominated by larger cleavage planes, which is consistent with a more brittle failure mode. Conversely, a low energy input (0.334 kJ/mm) increased the risk of insufficient fusion, resulting in smooth, flaky fragments characteristic of brittle failure. A moderate energy level (~0.390 kJ/mm) provided an optimal compromise, ensuring sufficient fusion while minimizing detrimental thermal effects, as evidenced by a homogeneous fracture morphology with a predominance of finer dimples. Furthermore, a larger gap geometry (1 mm) complicated process control, leading to an uneven distribution of filler material and a non-uniform fracture topography with local stress concentrators.

### 3.5. Microhardness Values

**Figure 12:**
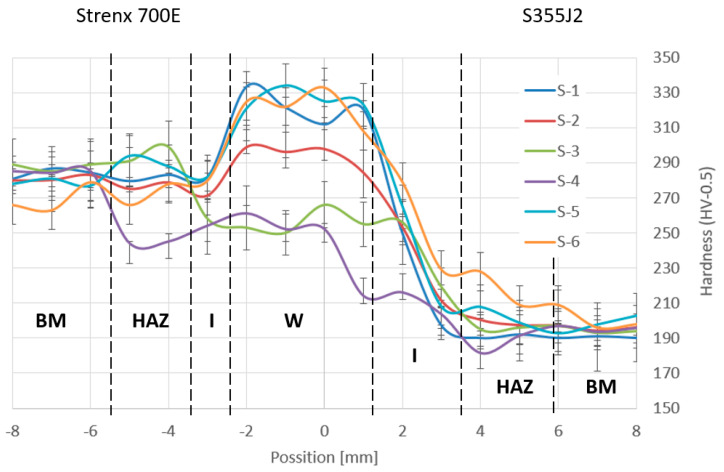
Microhardness distribution in the analyzed butt joint: BM—base metal, HAZ—heat area zone, I—insert, W—weld.

In the next step, microhardness tests were carried out on the joint cross-section for the prepared butt joints using an HV05 (4.9 N) indenter. Measurements were taken from the weld axis in both directions assuming a distance between points every 1 mm with the measurement line at the center of the thickness of the thinner of the materials being joined. The results obtained are shown in Figure 12.

The absence of a significant hardness through in the HAZ of the Strenx 700E for sample S3 suggests that the low heat input successfully mitigated the overtempering that typically weakens this region. These observed hardness profiles strongly support the previous conjecture regarding microstructural changes due to welding energy and show a narrower and less softened HAZ for low-energy welds, especially in the Strenx 700E side.

The undoubted advantage of lowering the welding parameters to a level that ensures the production of a correct weld and satisfactory strength is that it reduces the energy intensity of the process and, at the same time, the microhardness in the heat-affected zone. This provides tangible economic as well as exploitation benefits, as it reduces the risk of brittle fracture in the weld area.

On the basis of the tests carried out, it can be clearly stated that the appropriate preparation of the components before welding is important and relevant from the point of view of adhesion strength. In the case of positioning the workpieces with a 0.5 mm gap and pre-cleaning (sample S3) and with the welding torch at an angle to the materials to be joined, the highest mechanical strength of the joint of 610 MPa was obtained, which gives the possibility of transferring a load 40% higher than in the case of making a similar joint without pre-cleaning the surface (sample S4).

These results confirm that adequate preparation of the component surface before welding directly affects the quality and strength of the completed joint. Efforts should therefore be made to ensure that the materials, at least in the area where the joint is to be made, are de-skinned mechanically, e.g., by sandblasting or shot-blasting.

Analyzing the effect of the size of the welding gap when joining dissimilar materials (S6 and S7) on the strength of the butt joint, it can be seen that doubling the gap from 0.5 mm to 1 mm reduced the butt joint strength by 22% while increasing its ductility expressed in elongation corresponding to the maximum force by 73%.

## 4. Conclusions

This study successfully demonstrates that robust, load-bearing butt joints between dissimilar steels with a twofold difference in strength (S355J2 and Strenx 700E) are achievable for lightweight structural applications, provided process parameters are optimized. The investigation, focusing on CMT welding, yielded several important findings:

- Mechanical cleaning of joint surfaces prior to welding was the most influential factor, increasing joint strength by approximately 32%, presumably by eliminating inclusions and pores that act as fracture initiators.
- A reduced linear energy of 0.334 kJ/mm minimized the heat-affected zone (HAZ), prevented significant softening in the high-strength Strenx 700E steel, leading to superior tensile properties and more uniform deformation.
- The proposed thickness selection formula (Equation (1)), coupled with a minimal joint gap (0.5 mm), promoted even stress distribution and homogeneous plastic deformation across the dissimilar joint, emulating the desirable behavior of a monolithic material.
- Consequently, the parameter set designated as Sample S3, featuring mechanically cleaned surfaces, a 0.5 mm gap, and a low linear energy of 334 kJ/mm, is established as the optimum. This configuration delivers a balance of high strength (UTS of 616 MPa), adequate ductility, and minimal thermal distortion, directly addressing the core challenge of joining dissimilar materials.

The validated methodology and specific parameters presented provide a reliable framework for industrial fabrication. By enabling the production of high-integrity dissimilar joints, this work directly supports the design and manufacture of lightweight mechanical structures for demanding sectors such as commercial transportation and heavy machinery, where weight reduction must not compromise durability or safety.

## Figures and Tables

**Table 1:** The strength properties of the materials to be joined.

Steel Grade	S355J2	Strenx 700E
σ_y_ [MPa]	355	700
σ_UTS_ [MPa]	520	1100
A_5_ [%]	22	7
Hardness [HB]	220	290

σ_y_—yield stress [MPa]; σ_UTS_—ultimate tensile stress [MPa]; A_5_—elongation [%].

**Table 2:** Chemical composition of combined materials (wt.%).

Steel Grade	C	Si	Mn	P	S	Al	Cr	Cu	Ni	Mo	B	Nb	V	Ti
Strenx 700E	0.11	0.17	2.0	0.018	0.088	0.019	-	-	-	-	-	0.065	0.10	0.11
S355J2	0.2	0.35	1.5	0.03	0.025	0.016	0.25	0.019	0.2	-	-	-	-	-

**Table 3:** Welding process parameters of test joints of dissimilar materials.

Sample [S355J2 #5 + Strenx700E #3]								
Parameter	Value	S1	S2	S3	S4	S5	S6	S7
Surface preparation	C-cleaned	C			R			
	R-raw							
Gap (distance between joined elements)	mm	0.5						1
Current	A	140						
Voltage	V	22.9	17.4	14.4	14.4	17.4	22.9	22.9
Type of current		DC+						
Welding speed	m/min	0.35	0.30	0.29	0.29	0.30	0.35	0.35
Wire feed speed	m/min	4.9	3.7	3.9	3.9	3.7	4.9	4.9
Binder material		Lincoln Supramig HD ISO 14341-A-G46 4 M21 [25]						
Binder diameter	mm	1						
Shielding gas		M23 (90% Ar; 5% C02; 5% 02) ISO 14175 [26]						
Gas flow rate	L/min	14–16						
Welding energy Q_W_	kJ/mm	0.440	0.390	0.334	0.334	0.390	0.440	0.395
Welding method		CMT						
Angle torch	deg	30						

**Table 4:** Strength parameters characterizing the analyzed welded joints.

Specimen	*E_a_*	*E_a_*-Avg	Standard Deviation	Standard Error	*YS_a_*	*YS_a_*-Avg	Standard Deviation	Standard Error	*UTS_a_*	*UTS_a_*-Avg	Standard Deviation	Standard Error	*A_a_*-*UTS*	*A_a_*-*UTS*-avg	Standard Deviation	Standard Error
	[GPa]				[MPa]				[MPa]				[%]			
S1	174	176	2.160	1.247	340	338	1.633	0.943	472	462	14.855	8.576	2.80	2.80	0.008	0.005
	179				336				441				2.81			
	175				338				473				2.79			
S2	158	155	2.160	1.247	387	386	2.160	1.247	489	481	8.813	5.088	1.68	1.69	0.017	0.010
	154				383				486				1.71			
	153				388				469				1.67			
S3	178	180	1.633	0.943	457	459	2.082	1.202	634	616	15.706	9.068	4.84	4.85	0.022	0.012
	182				462				596				4.88			
	180				459				620				4.83			
S4	178	176	1.414	0.816	339	322	16.422	9.481	383	369	10.646	6.146	3.37	3.36	0.010	0.006
	175				328				357				3.35			
	175				300				369				3.35			
S5	161	162	1.414	0.816	254	260	4.899	2.828	337	334	6.952	4.014	2.20	2.21	0.017	0.010
	164				260				324				2.23			
	161				266				340				2.19			
S6	143	144	1.414	0.816	230	233	2.944	1.700	365	361	6.137	3.543	2.10	2.11	0.008	0.005
	146				237				352				2.11			
	143				232				365				2.12			
S7	110	112	1.633	0.943	186	184	1.291	0.745	291	282	7.789	4.497	3.22	3.23	0.008	0.005
	114				184				272				3.22			
	112				183				283				3.23			

*E_a_*—Apparent stiffness of the welded joint; *YS_a_*—Apparent yield strength of the welded joint; *UTS_a_*—Apparent strength of the welded joint; *A_a_*-*UTS*—Apparent elongation of the welded joint.

**Table 5:** Characteristic butt joint zones made of different materials using CMT welding.

Characteristic Butt Joint Zones	*E* [kJ/mm]	Cleaned Samples	Raw Samples
welded joint	0.334	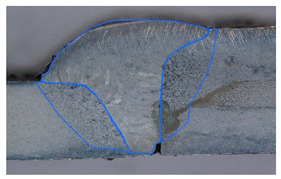	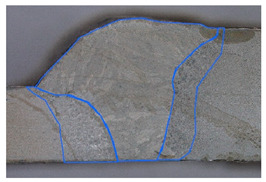
Area of W + I		16.386 [mm^2^]	18.722 [mm^2^]
Area of HAZ Strenx700E		4.699 [mm^2^]	4.535 [mm^2^]
Area of HAZ S355J2		5.137 [mm^2^]	4.612 [mm^2^]
welded joint	0.390	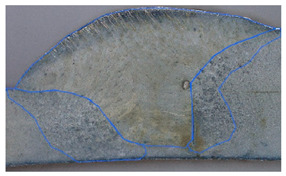	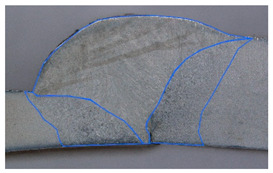
Area of W + I		24.912 [mm^2^]	24.614 [mm^2^]
Area of HAZ Strenx700E		5.500 [mm^2^]	7.069 [mm^2^]
Area of HAZ S355J2		7.186 [mm^2^]	9.805 [mm^2^]
Welded joint	0.440	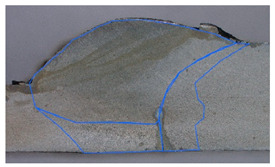	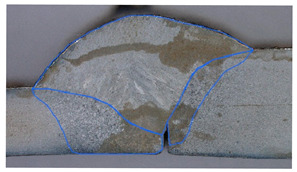
Area of W + I		29.344 [mm^2^]	26.751 [mm^2^]
Area of HAZ Strenx700E		6.216 [mm^2^]	4.54 [mm^2^]
Area of HAZ S355J2		7.296 [mm^2^]	9.588 [mm^2^]

**Table 6:** Coefficient and exponent of the monotonic strengthening curve of a welded joints.

Hollomon Strengthening Curve Parameters of Welded Joints			R-O Strengthening Curve Parameters of Welded Joints	
Sample	*K* [MPa]	*n*	*K*’	1/*n*’
S1	681	0.099	343	0.188
S2	616	0.127	415	0.147
S3	462	0.079	377	0.201
S4	395	0.097	548	0.143
S5	920	0.111	328	0.202
S6	733	0.095	251	0.273
S7	615	0.142	330	0.206

**Table 7:** The exponent of the strengthening curve according to the relation H.N. Hill.

Sample	The Exponent of the Strengthening Curve *n*_H_ (Equation (6)) of Welded Joints
S1	7.64
S2	6.01
S3	5.33
S4	5.64
S5	6.10
S6	6.23
S7	5.03

## Data Availability

The original contributions presented in this study are included in the article. Further inquiries can be directed to the corresponding author.

## Abbreviations

The following abbreviations are used in this manuscript:

CMT	Cold Metal Transfer
GMAW	Gas Metal Arc Welding
DED	Direct Energy Disposition
DIC	Digital Image Correlation
FSW	Friction Stir Welding
HAZ	Heat-Affected Zone
MAG	Metal Active Gas
MIG	Metal Inert Gas
RO	Ramberg-Osgood
TIG	Tungsten Inert Gas
UHSS	Ultra High Strength Steel
UTS	Ultimate Tensile Strength
YS	Yield Strength
HB	Brinell Hardness

## Appendix A

Detailed monotonic tensile test results of each specimens were presented in Figure A1, Figure A2, Figure A3, Figure A4, Figure A5, Figure A6 and Figure A7.
